# P-2069. Updated 2023–2024 (Monovalent XBB.1.5) COVID-19 Vaccine Effectiveness Against COVID-19–associated Hospitalization Among Adults — IVY Network, 20 U.S. States

**DOI:** 10.1093/ofid/ofae631.2225

**Published:** 2025-01-29

**Authors:** Jennifer DeCuir, Yuwei Zhu, Cassandra Johnson, Adam S Lauring, Emily T Martin, Manjusha Gaglani, Cristie Columbus, Ithan Peltan, Adit A Ginde, Nicholas Mohr, Kevin Gibbs, David Hager, Matthew Prekker, Amira Mohamed, Nicholas Johnson, Jay S Steingrub, Akram Khan, Laurence Busse, Lilian Lau, Steven Chang, Christopher Mallow, Jennie H Kwon, Nathan Shapiro, Ivana Vaughn, Basmah Safdar, Jarrod Mosier, Sascha Ellington, Fatimah S Dawood, Wesley H Self, Diya Surie

**Affiliations:** Centers for Disease Control and Prevention, Atlanta, GA; Vanderbilt University, Nashville, Tennessee; Vanderbilt University Medical Center, Oakdale, Minnesota; University of Michigan, Ann Arbor, MI; University of Michigan, Ann Arbor, MI; Baylor Scott & White Health, Temple, TX; Baylor Scott & White Health - Dallas, Dallas, Texas; Intermountain Medical Center and University of Utah, Salt Lake City, Utah; University of Colorado, Aurora, Colorado; University of Iowa, Iowa City, Iowa; Wake Forest University Baptist Medical Center, Winston-Salem, North Carolina; Johns Hopkins University School of Medicine, Baltimore, Maryland; Hennepin County Medical Center, Minneapolis, Minnesota; Montefiore medical center, Bronx, New York; University of Washington School of Medicine, Seattle, Washington; Baystate Medical Center, Springfield, Massachusetts; Oregon Health & Science University, Portland, Oregon; Emory University, Atlanta, Georgia; Stanford University, Cupertino, California; Ronald Reagan UCLA Medical Center, Los Angeles, California; University of Miami, Miami, Florida; Washington University School of Medicine, Saint Louis, MO; Beth Israel Deaconess Medical Center, Boston, Massachusetts; Henry Ford Health, Detroit, Michigan; Yale University School of Medicine, New Haven, Connecticut; University of Arizona, Tucson, Arizona; CDC, Atlanta, Georgia; Centers for Disease Control and Prevention, Atlanta, GA; Vanderbilt University Medical Center, Oakdale, Minnesota; Centers for Disease Control and Prevention, Atlanta, GA

## Abstract

**Background:**

On September 12, 2023, the Advisory Committee on Immunization Practices recommended updated 2023–2024 (Monovalent XBB.1.5) COVID-19 vaccination for all persons aged ≥6 months to prevent COVID-19. Few data are available on the effectiveness of updated 2023–2024 COVID-19 vaccine against COVID-19–associated hospitalization among adults, particularly in high-risk groups, including older adults, persons with comorbidities, and immunocompromised persons.

**Figure:**
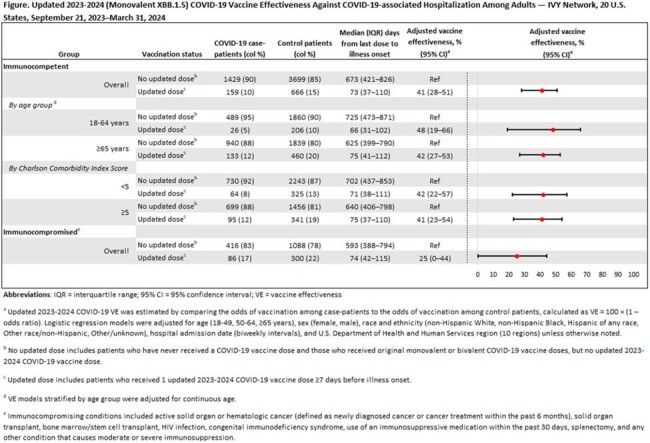

**Methods:**

Data from the Investigating Respiratory Viruses in the Acutely Ill (IVY) Network were used to conduct a case-control analysis estimating updated 2023–2024 COVID-19 vaccine effectiveness (VE) against COVID-19–associated hospitalization. During September 21, 2023–March 31, 2024, adults aged ≥18 years with COVID-19-like illness were enrolled at 26 hospitals in 20 U.S. states. COVID-19 case patients tested positive for SARS-CoV-2 by a nucleic acid or antigen test within 10 days of illness onset, while control patients tested negative for SARS-CoV-2. VE was estimated using multivariable logistic regression comparing the odds of receipt of an updated 2023–2024 COVID-19 vaccine dose versus no updated dose among case and control patients. VE models were adjusted for age, sex, race and ethnicity, admission date, and geographic region. Results were stratified by age, Charlson Comorbidity Index (CCI) score, and immunocompromised status.

**Results:**

A total of 7843 adults were enrolled, including 2090 COVID-19 case patients and 5753 control patients. Among immunocompetent adults, VE against COVID-19–associated hospitalization was 41% (95% CI=28%–51%, median time since updated dose = 73 days) among persons aged ≥18 years, 48% (95% CI=19%–66%) among persons aged 18–64 years, 42% (95% CI=27%–53%) among persons aged ≥65 years, 42% (95% CI=22%–57%) among persons with CCI < 5, and 41% (95% CI=23%–54%) among persons with CCI ≥5 (Figure). Among immunocompromised adults, VE against COVID-19–associated hospitalization was 25% (95% CI=0%–44%, median time since updated dose = 74 days).

**Conclusion:**

Updated 2023–2024 COVID-19 vaccination provided protection against COVID-19–associated hospitalization among immunocompetent adults. VE was similar across age groups and CCI scores, but may be lower among immunocompromised adults.

**Disclosures:**

Adam S. Lauring, MD, PhD, Roche: Advisor/Consultant Ithan Peltan, MD, Bluejay Diagnostics: Grant/Research Support|Regeneron: Grant/Research Support Nicholas Mohr, MD, Endpoint Health: Grant/Research Support Steven Chang, MD, PhD, Kiniksa: Consulting fee Christopher Mallow, MD, Medical Legal Consulting: Advisor/Consultant Ivana Vaughn, PhD, eMax Health Systems, LLC: Grant/Research Support|Evidera, Inc: Grant/Research Support

